# Foot health education for people with rheumatoid arthritis: the practitioner's perspective

**DOI:** 10.1186/1757-1146-5-2

**Published:** 2012-01-10

**Authors:** Andrea S Graham, Alison Hammond, Anita E Williams

**Affiliations:** 1 Centre for Health, Sport and Rehabilitation Research, University of Salford, Frederick Road, Salford, UK; 2 Directorate of Prosthetics, Orthotics and Podiatry, University of Salford, Frederick Road, Salford, UK

## Abstract

**Background:**

Patient education is considered to be a key role for podiatrists in the management of patients with rheumatoid arthritis (RA). Patient education has undoubtedly led to improved clinical outcomes, however no attempts have been made to optimise its content or delivery to maximise benefits within the context of the foot affected by rheumatoid arthritis. The aim of this study was to identify the nature and content of podiatrists' foot health education for people with RA. Any potential barriers to its provision were also explored.

**Methods:**

A focus group was conducted. The audio dialogue was recorded digitally, transcribed verbatim and analysed using a structured, thematic approach. The full transcription was verified by the focus group as an accurate account of what was said. The thematic analysis framework was verified by members of the research team to ensure validity of the data.

**Results:**

Twelve members (all female) of the north west Podiatry Clinical Effectiveness Group for Rheumatology participated. Six overarching themes emerged: (i) the essence of patient education; (ii) the content; (iii) patient-centred approach to content and timing; (iv) barriers to provision; (v) the therapeutic relationship; and (vi) tools of the trade.

**Conclusion:**

The study identified aspects of patient education that this group of podiatrists consider most important in relation to its: content, timing, delivery and barriers to its provision. General disease and foot health information in relation to RA together with a potential prognosis for foot health, the role of the podiatrist in management of foot health, and appropriate self-management strategies were considered to be key aspects of content, delivered according to the needs of the individual. Barriers to foot health education provision, including financial constraints and difficulties in establishing effective therapeutic relationships, were viewed as factors that strongly influenced foot health education provision. These data will contribute to the development of a patient-centred, negotiated approach to the provision of foot health education for people with RA.

## Background

Foot deformity and the associated symptoms of pain and stiffness are common in people with rheumatoid arthritis (RA), with up to 80% reporting pain at some point during the disease course [1,2]. Patient education is recommended as an integral part of the treatment regimen in RA [3]. Increased self-management through patient education is associated with improved clinical outcomes [4]. Patient education can range from simple information given as part of care, to more complex cognitive-behavioural education programmes that aim to support patient adherence to treatment [4].

Patient education is considered to be a key role for podiatrists in the management of people with RA [5,6]. Providing information relating to the purpose and use of clinical interventions, such as foot orthoses and specialist footwear, has the potential to improve patient adherence [7]. Using a patient-centred approach in the design and delivery of self-management programmes for foot health has been proven to be effective [8]. However, the most appropriate content of and delivery strategies for foot health patient education have not been investigated [9]. Refining these could improve foot health outcomes. How this education is delivered by podiatrists working with people with RA is also unknown.

Therefore, the aim of this study was to identify the nature and content of podiatrists' foot health education for people with RA. Any potential barriers to its provision were also explored.

## Methods

### Design

A focus group was conducted, as this is the most pragmatic approach for exploring attitudes, perceptions and ideas in this new area of research [10]. Individual interviews, whilst equally appropriate for ideas generation, do not have interaction between focus group participants, which promotes both consensus and clarifying diverse views between individuals [11]. The audio dialogue was digitally recorded and transcribed verbatim. A thematic framework was used to analyse the data, allowing the researcher to illustrate the main themes within a piece of text and enabling the transparent, methodical systematisation of textual data. To achieve this, a six stage process was used involving: coding the text; theme identification; thematic network construction; description and exploration of networks; summarisation of networks; and pattern interpretation [12].

### Participants

Participants were purposively recruited from Rheumatology Podiatry Clinical Effectiveness Group members working in National Health Service (NHS) Trusts across the north west region of England. The participants had to be qualified podiatrists, experienced in managing patients with RA, able to speak and read English and provide written consent. The proposed sample size was 7 to 12 participants, which is considered the optimum size for focus group interviews [10,13]

### Procedures

**Figure 1:**
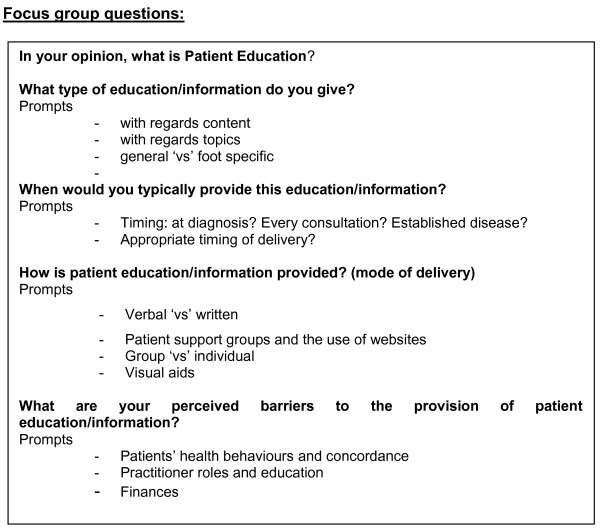
**Focus group questions: figure 1 gives details of the questions used to generate participant discussion during the course of the podiatrists' focus group**.

Ethical approval for the study was obtained by the University of Salford Research Ethics Committee and written informed consent was obtained from all participants prior to recruitment. The focus group questions were devised by the first author (AG), based on a review of the literature and contributions from the other two authors, one with patient education expertise (AH) and one with qualitative research expertise (AW). The questions were open-ended and designed to instigate in-depth discussion between the group participants across five sub-topics relating to the provision of foot-health education [Figure 1].

The focus group took place at the University of Salford as part of a regular scheduled meeting of the Rheumatology Podiatry Clinical Effectiveness Group. It was facilitated by the first author (AG) and field-notes taken by one of the other authors (AW). Any unanticipated topic areas were followed up with more questions by the first author. The dialogue was recorded digitally, transcribed verbatim by the first author and returned to the participants for verification and to support the trustworthiness of the data [14,15].

### Data analysis

The verified transcription of the dialogue was subject to thematic analysis [13] and categorised into 'Basic' and 'Organising' themes [Table 1]. Agreement for this categorisation was achieved between the first author (AG) and one of the other authors (AW) for both the thematic analysis and the data extracted [14,15]. Exemplars from the dialogue were extracted to demonstrate truthfulness of the data within each theme.

**Table 1:** Outline of the basic and organising themes developed from the thematic analysis

Basic Themes	Organising Themes
• Information Provision	
• Empowerment	The Essence of Patient Education

• Disease Diagnosis, Process & Prognosis	
• Interventions	
• Role of the Podiatrist	Content - the what and why
• Assessments	
• Non-podiatry related topic	

• General 'vs' specific education	
• Timing	A patient centred approach to content and timing

• External barriers to provision - organisational	
• Psychosocial barriers	Barriers to provision of Patient education
• Education with regards professional roles	
• Professional experience	
• Impact of patient concordance	

• The impact of patient knowledge	
• The impact of patient attitudes	
• The impact of practitioner attitudes	The Therapeutic Relationship
• The influence of age & gender	
• Role/title confusion	
• 'Taboo' subject areas	

• Group 'vs' individual provision	
• Verbal & written material	'Tools of the Trade'
• Audio-visual material	
• Web-based resources	

## Results

Twelve participants consented to participate. All had experience in managing people with RA and ranged from newly qualified podiatrists with an interest in working with patients with RA to those with experience within a Rheumatology multidisciplinary team. The average number of years since qualification within the group was 17.8 (SD = 9.8). Newly qualified podiatrists would have experience of working with people with RA across all undergraduate levels of clinical study and to a lesser extent, after qualification as an autonomous practitioner. Those working within the multidisciplinary team (n = 5) in acute services were more likely to work with consultant rheumatologists and specialist nurses. Those working in Primary Care Trust services (n = 7) had limited contact with a rheumatology multidisciplinary team.

Six organising themes emerged from the data analysis. Participants' names have been replaced with a pseudonym to ensure anonymity and confidentiality.

### Theme 1: The essence of patient education

This theme describes the participants' perception of patient education as a mechanism for patient empowerment. They considered that the process of information giving can impart the 'power' to patients to make appropriate decisions about consent and self-management. When asked what patient education *is*, the responses were short and to the point such as:

*(Patient education allows) "...Informed consent so that they can participate in the management regime" [Maria]*.

Patient education was considered useful for guiding patients according to their individual needs, and as Lisa stated, some of the content may not even be related to their feet:

"... *if they've got a question, you can say "well here's where you need to go and find out," you can put them in the right direction with the right agency. It's not even necessarily all about podiatry. Sometimes it's just helping them to find a way."*

The podiatrist's role as a point of access to other services that patients may not know about in relation to their specific health care needs was clearly thought of as a component of patient education.

### Theme 2: Content - what and why?

The participants considered that patients wanted general information. This included: how the disease and the drugs used to manage it, would impact upon their foot health; signs and symptoms relating to foot health that should prompt them to seek immediate advice from a healthcare professional; and the potential changes to their foot health as the disease progresses. Jane articulated that patients need:

"...*general information if they haven't got a specific problem, about foot health, about the impact of the drugs on their foot health and what sort of things (stops and thinks)... preventative advice..." [Jane]*.

There was a strong view that patients needed an explanation about foot health interventions and how they can help foot symptoms. As 'Ann' highlighted:

"*If they need orthotics then you've gotta do all kinds of explanations as to why they need them and how it's gonna help them, and then of course it's gonna be footwear to accommodate the orthotics. So I may have to explain y'know why you're doing... and find out y'know what they're willing to go along with..."*

The participants were often asked to provide information and advice that did not directly relate to foot health. This included the need for support for intimate personal issues, how to access welfare and support services and health promotion, such as smoking and alcohol consumption. The participants viewed this as a holistic approach to patient education:

*"I asked a patient about alcohol consumption... and was told like, seven pints, but he said it was every night... all sort of things came out of that. It was just a question I was asking, he was talking about methotrexate, medication ..." [Sara]*.

Informing patients about the role of the podiatrist was viewed with equal importance as providing foot health advice, in order to support patients in foot health self-management and in some cases, to ensure patient attendance at appointments with a podiatrist:

*"Patients turned up and they didn't know what they had been referred for. Or they weren't turning up and it was because they didn't know what they'd been referred for" [Ann]*.

The content of patient education was primarily not only to ensure that patients are aware of the disease, it's impact on lower limb health and the podiatrists' role, but also the medical management of RA, and the physical, social and personal issues associated with it.

### Theme 3: Patient-centred approach to content and timing of patient education

The content of patient education was influenced by: the patients' individual needs; disease status; age; and expectations of what podiatry can offer. The information provided was either general, such as basic foot health advice, or more specific, as identified by Jane:

*"I suspect at new diagnosis you're talking about the basics, how to manage general foot care (pauses).... general information if they haven't got specific foot problems... (pauses) I think early and late disease does have a slightly different slant on what you pick out as possibly more relevant at that point in time" [Jane]*.

The need for a patient-centred approach to foot-health education, that identifies the expectations of the patient, was articulated by Louise:

*"I think part of it [patient education] as well is patient expectations of what they're going to end up like..." [Louise]*.

This theme strongly illustrates the participants' view that foot health education cannot be overly prescriptive in its content and that timing needs to take into account the patient's defined needs.

### Theme 4: Barriers to provision of education

Other health practitioners' knowledge about the role of the podiatrist was thought to impact on the timely referral for foot care. As Jane highlighted:

"*Even if patients complain, the likelihood of actually getting looked at, y'know at new diagnosis... People just don't understand what it is we can do." [Jane]*.

The group thought that there should be a team approach to the provision of foot health education when patients are being managed within a multidisciplinary team, with a consensus as to what basic information all team members should be providing to avoid provision of detrimental and conflicting advice. However, foot health education provided by health practitioners, other than podiatrists, was viewed with scepticism by one participant:

"*That's a bit dodgy 'cos it's not always good." [Lisa]*.

Lack of time, due to overbooked clinics and a lack of finances with which to develop educational resources, were identified as further barriers to foot health education:

*".. and the numbers, the numbers of patients. It's very numbers-orientated in the acute [trust] (pauses)..... there's no money for leaflets [development]!' [Louise]*.

Patients' lack of understanding or acknowledgement that they need to change health behaviour was seen as an essential barrier to overcome in order to improve foot health. The 'domestic burden' of the patients' home circumstances, with other family members' needs being prioritised, or a poor financial status, were also viewed as barriers to patients following foot health advice:

*"You're giving them good shoe advice but they can't follow through 'cos they can't afford it." [Ann]*.

The ability of the podiatrist to empathise with the patients' experiences and employ appropriate consultation skills was seen as another barrier, notably amongst new graduates:

"*When I was newly qualified I couldn't understand why they didn't want to help themselves to get the best outcome" [Julie]*.

The challenges encountered when patients 'play off' one professional against another led to the labelling of such patients as 'non-compliant', resulting in patient education that was ineffectual, with reduced motivation for its provision. Participants described the refinement of consultation skills as a process requiring practice in negotiating with patients considered ambivalent:

*"When you've got patients in that are just like "oh yeah, yeah..." like that when you are talking to them, I think that you've got to keep practising it, to be encouraged, otherwise you do get a little bit demoralised." [Gill]*.

This theme clearly highlighted barriers to foot health education provision as: poor timing of referral by other members of the multidisciplinary team, lack of resources, such as time and money; perceived low patient compliance; and inexperience of novice podiatrists.

### Theme 5: The therapeutic relationship

The development of the therapeutic relationship describes the dynamic that exists between patient and practitioner and, in this context, focuses on how it influences patient education. The participants considered that the 'educational' role of the podiatrist was subtly altered when they are no longer the primary resource for information but act as a filter for what is 'good' and 'bad' information gained from elsewhere:

"*It is hard, you do have to sometimes say to them that... anybody can put anything they like on the internet... they seem to believe that if it's there in print it's go to be right" [Gill]*.

The patients' attitudes to their disease, was an influential factor in the development of the therapeutic relationship. Participants felt that patients who were in 'denial' about their diagnosis, or did not have foot health issues on their 'agenda', should not have foot health education "thrust upon them". The participants thought that, for some patients, engaging in foot health related 'activity', such as attending group educational sessions, would reinforce the perception that they were 'sick'. This may negatively influence the relationship with the practitioner and the potential to change their health behaviour:

*"They don't want to become part of the 'rheumatology world' because 'I'm not one of the sick people' y'know? Which you can understand." [Lisa]*.

Practitioner attitudes appeared to impact on the provision of education during the consultation. The need to be 'firm' or 'compromising' with patients was described:

*"I try to make everything sound like a compromise now. Especially for women it has to be a compromise" [Julie]*.

Empathy between these female practitioners and their female patients appeared to influence the patient - practitioner relationship and thus the effectiveness of foot health education. It was considered that those of the same gender would be able to relate to each other more effectively. Discussion of 'difficult' subject areas (such as footwear style with female patients) influenced the participants' ability to relate to their patients:

"*We all like to wear high heels and nice shoes when we go out.... you have that empathy with them" [Nancy]*.

The public's perception of the podiatrist was viewed by the participants as an influencing factor on the patient - practitioner relationship. It was thought by the group that 'podiatrists' are typically viewed by patients as having a more specialised role, with 'chiropodists' having more basic expertise. This confusion over professional title, and hence expertise, can influence patients' expectations about the information they expect.

"*They [patients] have some concept that there is some difference between a podiatrist and a chiropodist, they say "you're not quite the same as that, what is it that you do?"' [Lisa]*.

A number of factors influence the therapeutic relationship including: the patients' level of foot health and disease knowledge prior to the initial consultation; the subtle change in the subsequent role of the podiatrist as an educator to re-educator; the patients' attitude to the disease; the age and gender of both the patient and the podiatrist; and the patients' confusion over the professional title.

### Theme 6: 'Tools of the trade'

This theme describes the methods most commonly used and the issues most relevant to the participants in the delivery of foot health education. Information provided in a one-to-one context, using written advice and visual aids (such as examples of moisturising products) to reinforce verbal advice, was most commonly used. Some used locally produced leaflets and some used other sources, such as footwear company catalogues and literature from charities (for example Arthritis Research UK). It was considered that care was needed when providing such written information, as the language used might be difficult for some patients to understand and could become a barrier to effective patient education. Directing patients in using the Internet appropriately was seen as additional supportive information, although this method was not used by all participants.

The combination of verbal and written information was viewed as important to enable the patient to reflect upon what had been said during the consultation and to act as a 'aide memoire':

"*You could provide verbal education on top of having a minimum to hand out and then they've had something to reflect on after their consultation. [Patients] tend to forget half of what you tell them anyway' [Meg]*.

Group education was considered useful in providing peer support for patients, reducing the feeling of isolation and as a conduit for the provision of general information. However, it was not widely used, due to a lack of: evidence for its' effectiveness; feasibility; patient motivation; and finance. One-to-one patient education was considered more useful as it provided more tailored, individualised information in an environment that might be more comfortable for patients to discuss personal issues:

"*'I think some people are just more comfortable on a one to one basis... it's quite a personal thing isn't it?" [Maria]*.

This theme illustrates the most widely used format for patient education is one-to-one verbal delivery, supported with written material.

## Discussion

The participants' views on patient education for people with RA are that it is a mechanism for facilitating foot health self-management and enabling informed consent for foot health interventions. The literature relating to foot health education in patients with diabetic foot problems [16] supports structured education and information giving to enhance self-efficacy and improve health behaviour.

The participants perceived that patients needed to know about RA, its cause and its impact on future foot health. Patients also want to know about symptoms requiring urgent attention and good self-care to prevent deterioration. These are the key topics any podiatrist should address, together with modifying lifestyle factors such as smoking and excessive alcohol consumption. These topics are recommended in the Podiatric Rheumatic Care Association Musculoskeletal Foot Health Standards [5]. Educating patients about such risk factors for cardiovascular disease is vital, given the association between RA and cardiovascular disease [17]. Podiatrists have the skills and knowledge to assess and monitor patients' lower limb vascular status and are well placed to discuss the effect of smoking on lower limb health, such as the development of peripheral arterial disease, which is accelerated in people with RA [18,19]. Patient education for people with RA about cardiovascular disease has been recognised as being poorly promoted by health care professionals [20].

It was strongly considered that the scope of practice of podiatrists in relation to managing people with RA is not widely recognised within the medical community or by patients. If patients and other members of the multidisciplinary team are unaware of what can be provided about foot health management, then timely and appropriate referral cannot be achieved. Members of the rheumatology multidisciplinary team need to be agreed as to the foot health education provided to patients in their service [5] to avoid conflicting information being given to patients. This issue reflects the need for podiatrists to educate other members of the multidisciplinary team about foot health. Ensuring that team members are fully conversant with each others' role within the wider management of people with RA may help to resolve this. Care pathways which detail traditional foot health interventions and educational needs of people with RA [6] can provide evidence-based guidance that supports all multidisciplinary team members in foot health management.

A perceived lack of awareness of the podiatrist's role by the members of the multidisciplinary team creates confusion. This was thought to be due to 'dual professional identity' resulting from the continued use of 'podiatrist' and 'chiropodist' as professional titles. The retention of the title 'chiropodist' reflects the original role of social foot-care [21] compared with the current role including lower limb assessment, independent diagnosis and extended skills such as steroid injection therapy and non-medical prescribing.

Health education provision for people with RA should be flexible, timely and patient-centred [22,23]. The participants expressed that foot health education content should be tailored according to individual need, disease stage, age, gender and recognition of ability to engage in positive health behaviour. The trans-theoretical model of behavioural change [24] is acknowledged as being a useful tool for identifying a persons' readiness to make changes in health behaviour [25]. The participants identified the need to '... move patients from the stage of pre-contemplation to contemplation' in order to effect positive behaviour change.

Motivational interviewing techniques [26] can be highly effective in engaging patients in change talk, though the use of these techniques is a skill in itself. The lack of such skills was identified as a potential barrier to the provision of foot health education, particularly in those who were more recently qualified and who had less clinical experience. Participants felt well prepared by their undergraduate training in terms of understanding the underlying theory of motivational interviewing techniques, but in the 'real world' their expectations had been lowered through experience of patients who 'did not want to help themselves by complying with foot health advice'. Perhaps the challenge here lies in equipping podiatrists with strategies to cope with patient resistance to changing health behaviour, alongside skills in effective patient-centred consultation. This should be provided within the undergraduate curriculum and as part of continuous professional development.

There is no consensus as to the most appropriate time to provide foot health education. Patients should have timely access to relevant foot health specific advice and information that enables them to recognise variations in disease activity, focussing on issues of particular relevance at any given time [5]. The use of one-to-one consultations that can be responsive to the patient's individual needs and provide a less intimidating environment is more appropriate in these circumstances. Further, practitioners should be mindful of the fact that not all patients desire or see the benefits of changes in health behaviour in the short term, but their perceptions may alter with time [25].

This study found that one-to-one delivery of foot health education during the consultation, combining verbal and written material was the most common method of delivery, with minimal use of group education and charity websites such as Arthritis Research UK and the National Rheumatoid Arthritis Society. There has been no direct comparison of one-to-one versus group education for people with RA. The use of group education can provide a supportive environment in which patients can discuss common issues together with the use of individualised verbal information supported by printed documents and reputable patient support group websites [22]. Further to this the implementation of educational behavioural programmes has been found to maintain benefits, such as improved pain scores and self-efficacy, for up to 12 months [27] and may prove cost-effective to the NHS in the long term [8]. However, this should be balanced with the potential additional 'cost-to-self' for patients, as this study highlighted that socioeconomic factors are thought to influence patients' ability to comply with certain aspects of foot health education such as the purchasing of appropriate footwear that may cost more than they would normally spend. There are currently no foot health education programmes that cater for people with RA, though the feasibility of patients with RA participating in a foot health self-management programme has been investigated [28]. At initial diagnosis patients may not be ready to participate in a comprehensive programme of foot health education, though this is yet to be ascertained.

This is the first study to explore podiatrists' perceptions of foot health education for people with RA. The views expressed within this study are restricted to podiatrists working within rheumatology who attend a Clinical Effectiveness Group (CEG) and were thus purposively selected. It could be argued that focus groups should consist of participants that do not know each other to avoid the influence of pre-existing relationships upon the outcomes of the discussion and promote a more honest response [29]. Further to this the presence of more experienced, senior practitioners within the group may have resulted in the modification of the responses from their junior or less experienced colleagues. However, the trust that can be found within members of groups who already know each other can be a positive and encouraging influence upon the discussion; participants may feel more able to challenge each other's views if they feel comfortable with each other [10,30]. A constant positive group dynamic was observed throughout this focus group, facilitating involvement of all participants in the discussion, without stifling the richness of data generated.

It is acknowledged that the use of other qualitative methods such as Interpretative Phenomenological Analysis [31] could reveal more complex interpretative aspects within this data. However, the use of thematic framework analysis in this study allows for a thematic description of the entire data set, which is appropriate for the investigation of this under-researched area and the identification of the most predominant themes [32].

The number of participants in this focus group could be viewed as relatively high, the ideal number being suggested as between 6 and 10 [10,29,30]. However, larger numbers can be used where it aligns with the research aims and the generation of concepts is required [33]. A similar argument may be applied to the number of focus groups conducted. Only one focus group was conducted and additional focus groups may have added to the data. However, there is no consensus as to the ideal number of focus groups that should be conducted, with the literature suggesting a single group [34] to over 50 groups [30]. Therefore, a pragmatic approach was adopted that considered the purpose of the study, the financial cost, time available and perceived attainment of data saturation.

The participants were from the northwest region of England, which may mean that the results are not generalisable. However, they were from a range of services and duration of clinical experience and so are likely to be representative of UK podiatrists. Future research into podiatrists' opinions of foot health education should involve both male and female practitioners, those from a wider geographical area and those in private practice. Additionally, a wider perspective that investigates the perceptions of other allied health practitioners and consultant rheumatologists in relation to the provision of foot health education may be of potential importance. The patients' perspective on their experiences and educational needs requires investigation from a wide geographical perspective.

The ultimate aim of future research should be the development of a patient-centred and negotiated approach to foot health education, through which the individuals' needs and preferences are identified.

## Conclusion

This study has identified aspects of patient education that this group of podiatrists found most influential in its delivery including; what they perceive the role of foot health education to be, the main content including general disease and foot health related information, appropriate strategies for self-management and the role of the podiatrist in managing the foot health of people with RA. The need for a tailored approach to delivery, according to the needs of the individual over the life span of the patient through identification of the patient's agenda, was highlighted as being influential in the development of an effective therapeutic relationship. Potential barriers to its delivery included a lack of patient-centred consultation skills, the financial status of the patient and the NHS trust and time constraints. From the podiatrists' perspective this identifies a need to develop foot health education that encompasses both the patients' needs and podiatrists' responsibilities. The ultimate aim of this would be to support self-efficacy and appropriate foot health behaviour, thereby improving the foot health for people with RA.

## Competing interests

The authors declare that they have no competing interests.

## Authors' contributions

AG conceived and executed the study design (with contributions from AW and AH), interpreted the findings with assistance from AW and drafted the manuscript with assistance from AW and AH. All authors read and approved the final manuscript.
